# Molecular epidemiology of canine parvovirus type 2 in Vietnam from November 2016 to February 2018

**DOI:** 10.1186/s12985-019-1159-z

**Published:** 2019-04-27

**Authors:** Minh Hoang, Wei-Hao Lin, Van Phan Le, Bui Thi To Nga, Ming-Tang Chiou, Chao-Nan Lin

**Affiliations:** 1 Department of Veterinary Medicine, College of Veterinary Medicine, National Pingtung University of Science and Technology, Pingtung, Taiwan; 2 Department of Anatomy and Histology, College of Veterinary Medicine, Viet Nam National University of Agriculture, Hanoi, Vietnam; 3 Animal Disease Diagnostic Center, College of Veterinary Medicine, National Pingtung University of Science and Technology, Pingtung, Taiwan; 4 Department of Microbiology and Infectious Disease, College of Veterinary Medicine, Viet Nam National University of Agriculture, Hanoi, Vietnam; 5 Department of Veterinary Pathology, College of Veterinary Medicine, Viet Nam National University of Agriculture, Hanoi, Vietnam; 6 Research Center for Animal Biologics, National Pingtung University of Science and Technology, Pingtung, Taiwan

**Keywords:** Canine parvovirus type 2, Genotype, SimpleProbe® real-time PCR, Vietnam, SimpleProbe®

## Abstract

**Background:**

Canine parvovirus type 2 (CPV-2) was first identified in the late 1970s; it causes intestinal hemorrhage with severe bloody diarrhea in kennels and dog shelters worldwide. Since its emergence, CPV-2 has been replaced with new genetic variants (CPV-2a, CPV-2b, and CPV-2c). Currently, information about the genotype prevalence of CPV-2 in Vietnam is limited. In the present study, we investigated the genotype prevalence and distribution of CPV-2 in the three regions of Vietnam.

**Methods:**

Rectal swabs were collected from 260 dogs with suspected CPV-2 infection from northern, central, and southern Vietnam from November 2016 to February 2018. All samples were identified as parvovirus positive by real-time PCR, and further genotyping was performed using a SimpleProbe® real-time PCR assay.

**Results:**

Of the 260 Vietnamese CPV-2 isolates, 6 isolates (2.31%) were identified as CPV-2a, 251 isolates (96.54%) were identified as CPV-2c and 3 isolates (1.15%) were untypable using the SimpleProbe® real-time PCR assay. In northern Vietnam, the percentages of CPV-2a and CPV-2c were 2.97% (3/101) and 97.3% (98/101), respectively. In central Vietnam, the percentages of CPV-2a and CPV-2c were 1.11% (1/90) and 98.89% (89/90), respectively. In southern Vietnam, the percentages of CPV-2a and CPV-2c were 3.03% (2/66) and 96.97% (64/66), respectively. CPV-2b was not observed in this study. The VP2 genes of CPV-2c in Vietnam are more genetically similar to those of CPV-2c strains in China and Taiwan than to those of prototype CPV-2c strains (FJ222821) or the first Vietnamese CPV-2c (AB120727).

**Conclusion:**

The present study provides evidence that CPV-2c is the most prevalent variant in Vietnam. Phylogenetic analysis demonstrated that the recent Vietnamese CPV-2c isolates share a common evolutionary origin with Asian CPV-2c strains.

**Electronic supplementary material:**

The online version of this article (10.1186/s12985-019-1159-z) contains supplementary material, which is available to authorized users.

## Background

Canine parvovirus type 2 (CPV-2) is one of the most dangerous enteropathogens, causing fatal disease in dogs and puppies worldwide [1]. CPV-2 is a nonenveloped, small DNA virus with a diameter of approximately 25 nm and a single-stranded DNA genome of approximately 5 kb [2]. CPV-2 belongs to the genus *Parvovirus* in the family Parvoviridae, which includes feline panleukopenia virus (FPV), mink enteritis virus, raccoon parvovirus, and porcine parvovirus [3]. Clinical manifestations of CPV-2 infection are characterized by intestinal hemorrhage with severe bloody diarrhea; other clinical signs include anorexia, depression and vomiting [1, 3, 4].

CPV-2 is a rapidly evolving virus, leading to the mutation of novel variants since its first recognition in the late 1970s [5]. For example, CPV-2a was approximately discovered in 1980 in the USA, and CPV-2b and 2c were identified in 1984 and 2000 in the USA and Italy, respectively, based on residue 426 (Asn in 2a, Asp in 2b, and Glu in 2c) in the VP2 protein of the parvovirus [4, 6]. When a novel CPV-2 variant appears, it very rapidly replaces old variants [7, 8]. In recent decades, CPV-2c has been found to be widespread in European countries [1, 9], the USA [10], South America [11–13] and Africa [14, 15]. The first reported occurrence of the CPV-2c variant in Vietnam was in 2004 [16]; however, since then, this strain has not been prevalent in Asia [17]. Surprisingly, novel Asian CPV-2c isolates were identified in China [18–21], Taiwan [17, 22], Laos [23] and Thailand [24] in the past few years.

In Vietnam, CPV-2 infection was first observed as sporadic cases in 1994 (unpublished data). Subsequently, after its first emergence, there were widespread outbreaks of canine hemorrhagic enteritis with high morbidity and mortality occurring across the whole country [16]. Along with the increasing number of pet dogs in Vietnam, CPV-2 infection has emerged as a veterinary public health concern that greatly affects puppies because of its high mortality and morbidity. However, current information related to the antigenic types of CPV prevailing in Vietnam is poorly understood. Thus, in the present study, we investigated the genotype prevalence and distribution of CPV-2 from naturally infected dogs in three regions of Vietnam using a SimpleProbe® real-time polymerase chain reaction (PCR) assay.

## Methods

### Specimen collection

Rectal swabs were collected from 260 dogs with suspected CPV-2 infection from northern, central, and southern Vietnam from November 2016 to February 2018. These dogs had displayed clinical symptoms of CPV-2 infection, including diarrhea or bloody diarrhea. Fecal samples were collected from dogs with alimentary signs (diarrhea or bloody diarrhea) by inserting a swab into the rectum (~ 2 cm), rotating the swab and then resuspending the swab in 1 ml of phosphate-buffered saline. All specimens were transported on ice and stored at -20 °C at the key laboratory for biotechnology and animal disease, Vietnam National University of Agriculture, Hanoi, Vietnam. Information including the sampling year, age, clinical history, and CPV-2 types of the sampled dogs is summarized in Additional file 1.

### DNA extraction and parvovirus screening

Total DNA was extracted using a Genomic DNA Mini Kit (Geneaid Biotech, Ltd., Taipei, Taiwan) following the manufacturer’s protocol. All of the clinical specimens were confirmed to be infected with parvovirus by real-time PCR, as described by Lin et al. [25].

### SimpleProbe® assay for CPV-2 genotyping

Parvovirus-positive samples were further characterized by a SimpleProbe® real-time PCR assay, following the method developed by Hoang et al. [26]. First, the reaction was carried out in a final volume of 10 μl containing 3 mM MgCl_2,_ 3 pM SimpleProbe® (TIB MOLBIOLGmbH, Berlin, Germany), 5 pM and 2 pM each of primers Parvo-F (5′-ACA CCT gAg AgA TTT ACA TAT ATA gCA CA-3′) and Parvo-A (5′-ATT AgT ATA gTT AAT TCC TgT TTT ACC TCC-3′), 1 μl 10x Light Cycler 480 Genotyping master mix and 1 μl 10x diluted template DNA. Real-time PCR was cycled under the same thermal conditions as those previously described for the SimpleProbe® assay [26].

### VP2 gene amplification, sequencing and sequence analyses

To clone full length VP2, we performed PCR amplification using the primer pair VP2F (5′- CGGTGCAGGACAAGTAAAA -3′)/VP2R (5′-GGTGCTAGTTGATATGTAATA -3′) that amplified a 1755 bp fragment of the gene encoding the capsid protein. The PCR conditions were as follows: denaturation at 94 °C for 2 min; 40 cycles of denaturation at 94 °C for 30 s, annealing at 50 °C for 1 min, and extension at 72 °C for 2 min; and a final extension step at 72 °C for 5 min. PCR products were electrophoresed, followed by the purification of electrophoresis products by a Wizard® SV Gel and PCR clean-up system (Promega Corporation, USA). The PCR products obtained from the previous step were cloned by a T&A® Cloning kit before being sent for automated sequencing (MB mission biotech, Inc., Taiwan). The resulting sequences were compared with reference FPV (M38246), CPV-2 (M38245), CPV-2a (M24003, and M24000), new CPV-2a (AY742953, AB054213, EU009200, JQ686671, KF676668, and KR611488), CPV-2b (M74852 and M74849), new CPV-2b (AB120720, AB120721, AB120722, AB120723, AB120724, AB120725, AB120726, AY742955, AY869724, JQ268284, KR611459, and KR611461), CPV-2c (FJ222821, AB120727, FJ005235, KM236569, KR611522, KT156832, KT162005, KY937650, MF467229, and KU244254), CPV-2a vaccine strain (FJ197847) and previous Vietnamese strains (AB120720, AB120721, AB120722, AB120723, AB120724, AB120725, AB120726). The Clustal W method and MegAlign program (DNASTAR, Madison, WI, USA) were used for multiple alignments of the nucleic acid and amino acid sequences. Phylogenetic analyses were conducted by the maximum likelihood methods using MEGA 6 based on the Tamura-Nei model [27].

## Results

### SYBR-based real-time PCR amplification

**Fig. 1:**
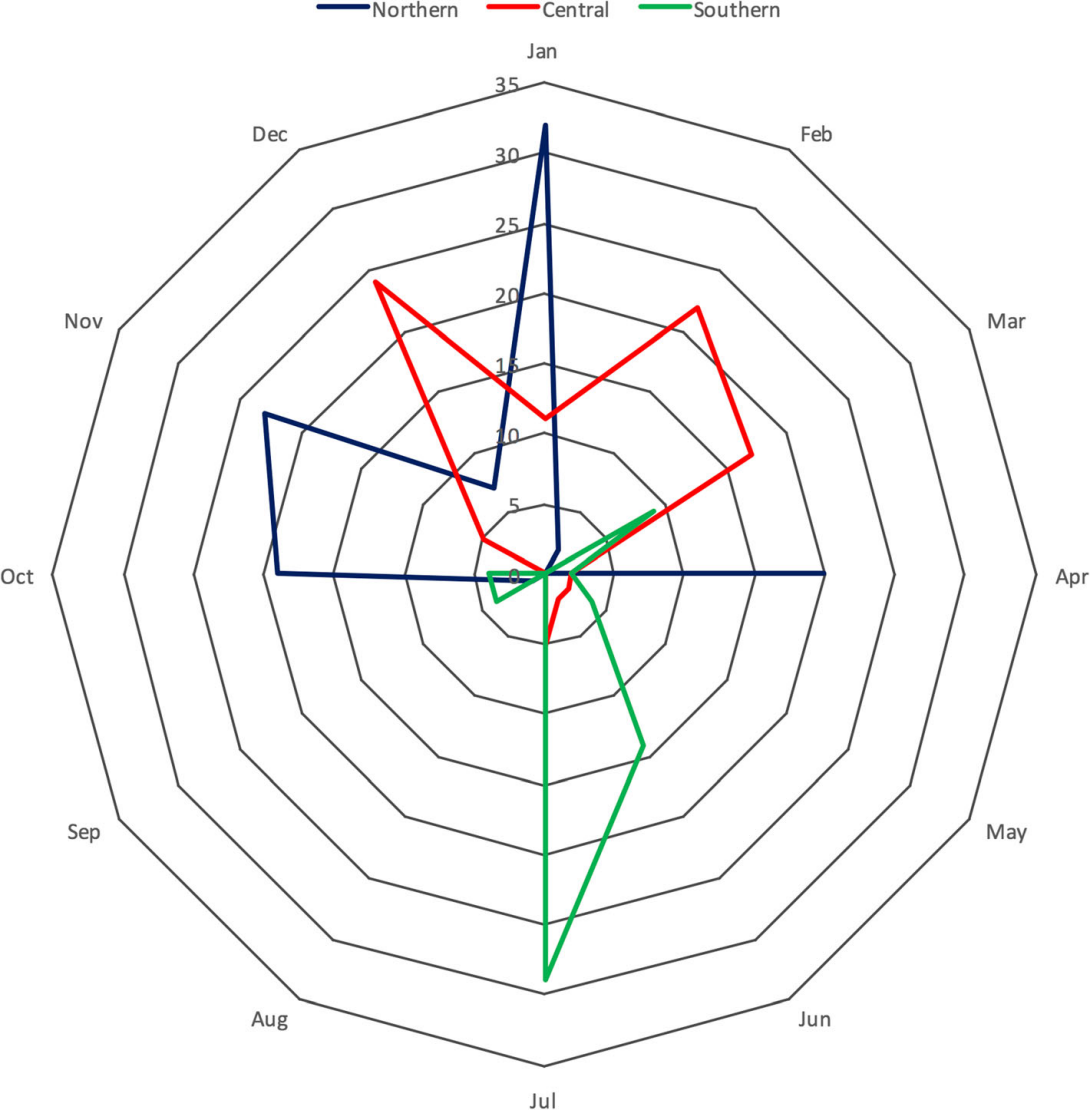
Monthly distribution of CPV-2 isolates in three regions of Vietnam. The numbers of positive cases in each of the three regions were individually calculated according to the sample collection date to analyze the monthly distribution

A total of 260 specimens were collected from suspected parvovirus-infected dogs in three regions of Vietnam, including northern, central and southern Vietnam. All 260 samples were positive for parvovirus DNA. The age of the sampled dogs ranged from 1 month to 12 months old. The demographic summary of puppies that tested positive for parvovirus by real-time PCR is also presented in Additional file 1. The number of puppies less than 3 months old accounted for 26.92% (70/260) of the sample, dogs of 3 to 6 months of age accounted for 49.62% (129/260) of the sample, and dogs older than 6 months accounted for 8.85% (23/260) of the sample. For 38 samples, the age group was unspecified. In terms of gender, 51.15% (133/260) of the sample was male dogs, and 36.15% (94/260) was female dogs. For 33 samples, the gender was not reported. In the northern areas of Vietnam, CPV-2 cases often peak in November and January, while the peak time for central areas is from December to March, and the peak time for southern areas is in June and July (Fig. 1).

### Genotype analysis and geographical distribution of CPV-2 variants

**Fig. 2:**
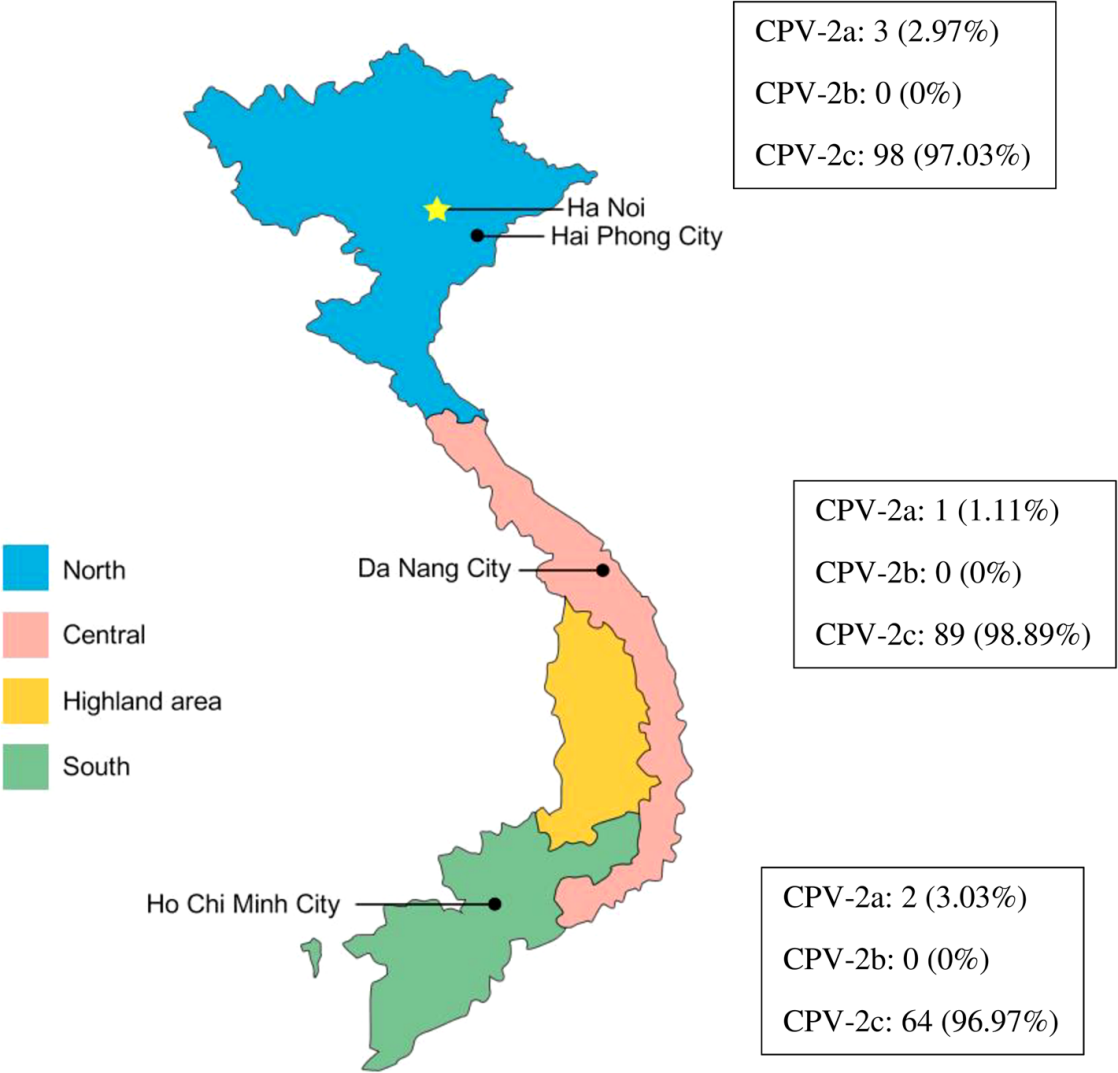
Geographical distribution of CPV-2 variants in three regions of Vietnam. Of a total of 257 genotyped CPV-2 isolates, 101 isolates, 90 isolates and 66 isolates were collected from northern, central and southern Vietnam, respectively. The percentage of CPV-2 variants was independently calculated in each region

**Fig. 3:**
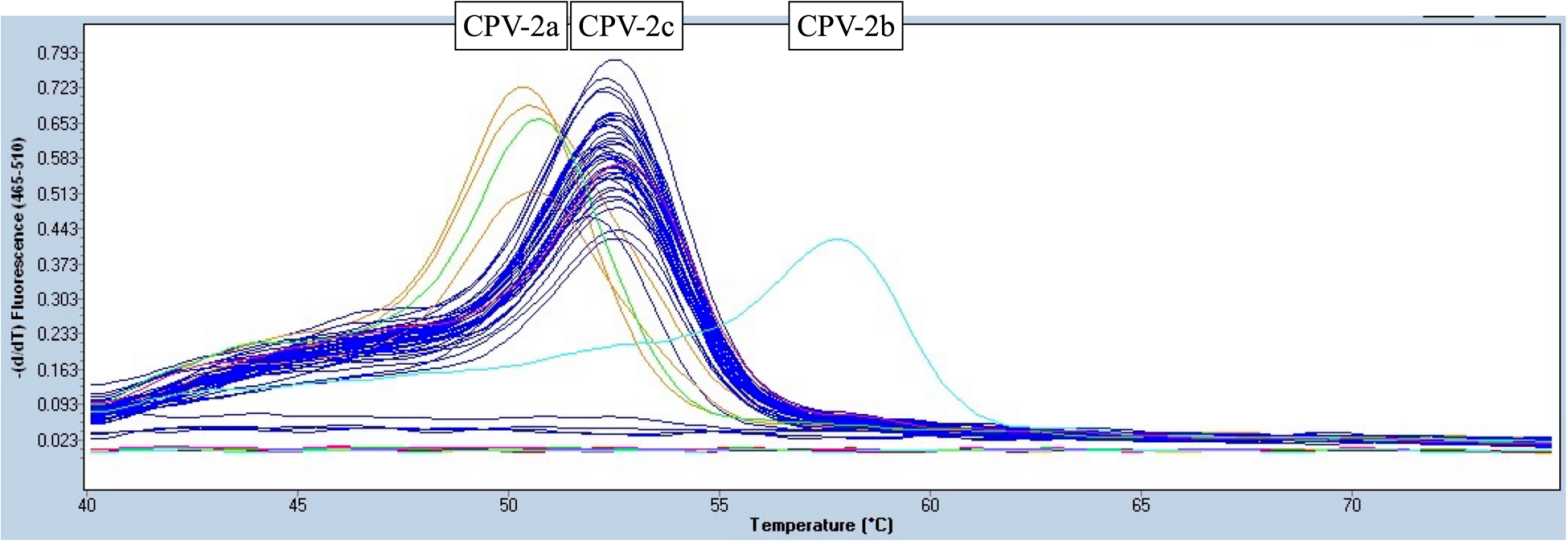
Melting curve analysis of clinical CPV-2 isolates using a SimpleProbe® real-time PCR assay

Among the 260 samples collected from the three regions in Vietnam, 257 samples (98.85%) were clearly genotyped according to melting temperature analysis using a SimpleProbe® real-time PCR assay (Table 1). However, there were three cases (1.15%) from northern Vietnam for which differentiation was not possible by the SimpleProbe® real-time PCR assay (Additional file 1). Among 257 samples, six isolates (2.31%) were identified as CPV-2a and 251 isolates (96.54%) were identified as CPV-2c. In northern Vietnam, the percentages of CPV-2a and CPV-2c were 2.97% (3/101) and 97.03% (98/101), respectively. In central Vietnam, the percentages of CPV-2a and CPV-2c were 1.11% (1/90) and 98.89% (89/90), respectively. In southern Vietnam, the percentages of CPV-2a and CPV-2c were 3.03% (2/66) and 96.97% (64/66), respectively (Fig. 2). CPV-2b was not observed in this study (Fig. 3). Our results showed that CPV-2c is currently the most prevalent CPV-2 field strain circulating in Vietnam.

**Table 1:** Melting temperature of Vietnam samples

CPV-2 genotype	SimpleProbe® analysis		Reference melting temperature (CI95%) [26]
	Number	Melting temperature of Vietnamese samples (CI95%)	
2a	6	50.23 (50.2–50.3)	50.2 (50.1–50.5)
2b	0	N/A^a^	57.8 (57.7–58.5)
2c	251	52.75 (52.7–52.8)	52.3 (52.2–53.2)

^a^*N/A* not available

### Genotype of CPV-2 vaccinated dogs

A total of 44 dogs (16.92%) were confirmed to be vaccinated against CPV-2. Among 44 CPV-2 vaccinated dogs, 6 were younger than 3 months old, and 32 were 3 months old or older; the age of the remaining 6 dogs could not be accurately determined. Interestingly, all vaccinated dogs were identified as having CPV-2c infection using the SimpleProbe® real-time PCR assay.

### Amino acid and nucleotide sequence analysis of VP2

**Fig. 4:**
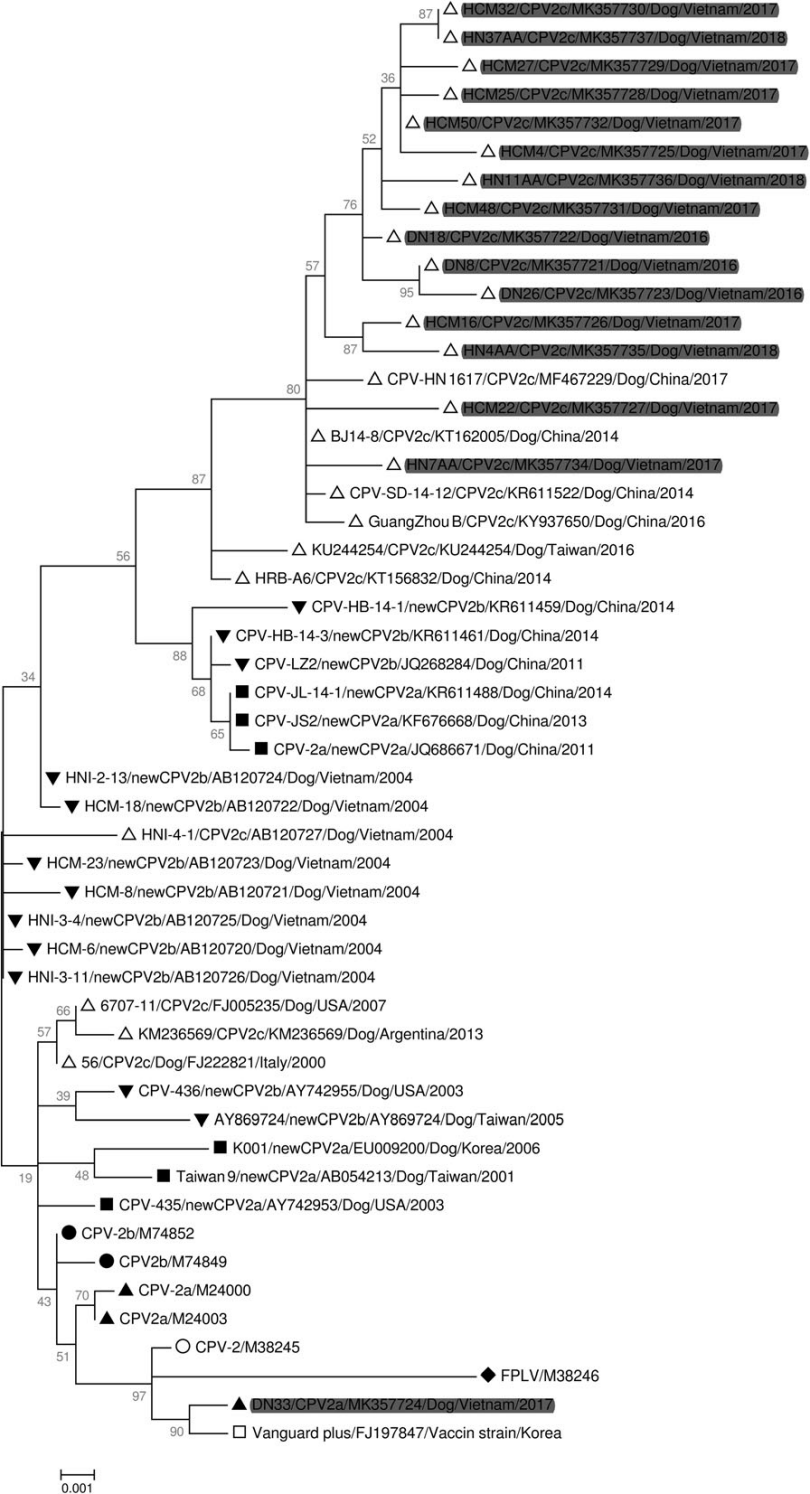
Phylogenetic relationships based on the complete VP2 gene of CPV-2 between**.** Vietnamese isolates and reference strains. HN: HaNoi (North Vietnam), DN: DaNang (Central Vietnam), HCM: HoChiMinh city (South Vietnam), CPV: Canine parvovirus, FPLV: Feline panleukopenia virus. The analysis was performed employing the maximum likelihood method based on 1000 replicates using MEGA 6 software. CPV variants are indicated by □, ◆, ◯, ▲, ■, ●, ▼ and △ for the vaccine strain, FPLV, CPV-2, CPV-2a, new CPV-2a, CPV-2b, new CPV-2b, and CPV-2c, respectively. Gray underlay represents CPV-2 isolates in the present study

To investigate the association and diversity of CPV-2 strains circulating in Vietnam, we sequenced and analyzed 16 complete VP2 genes of CPV-2 strains collected from the three regions of Vietnam. Phylogenetic analysis based on the nucleotide sequences of the complete VP2 gene revealed two clusters that were prototype CPV-2c strains and Asian CPV-2c clusters (15 isolates from this study together with the Chinese strain [KR611522, KT156832, KT162005, KY937650, and MF467229], and Taiwanese strain [KU244254]) (Fig. 4). The VP2 genes of CPV-2c in Vietnam are more genetically similar to CPV-2c strains in China and Taiwan than to prototype CPV-2c strains (FJ222821) or first Vietnamese CPV-2c (AB120727) (Fig. 4). In addition, one Vietnamese strain (DN33) in this study was clustered with a CPV-2a vaccine strain (FJ197847) (Fig. 4).

There were several nucleotide mutations in the VP2 gene of all strains (Table 2), but only 6 mutations (nucleotide position 13, 800, 899, 970, 1109, and 1341) resulted in changes in the amino acid sequence. Amino acid sequence comparison among the 11 reference strains and 16 isolates at amino acids 5–555 is illustrated in Table 2. For the complete VP2 analysis, one sequence with an Asn and 15 sequences with a Glu at position 426 were classified as CPV-2a and CPV-2c, respectively. All CPV-2c strains showed substitution at position Ala5Gly, Phe267Tyr, Tyr324Ile, and Gln370Arg (Table 2). Thirteen of the 15 CPV-2c strains obtained showed a unique substitution at position 447 (Ile to Met) caused by mutation of TAT to TGT at nucleotide positions 1340–1342 of the VP2 gene (Table 2).

**Table 2:** Nucleotide sequences and amino acid substitution in the full VP2 gene of CPV-2

Strains	Position of nucleotide sequence (amino acid)													
	13–15 (5)	259–261 (87)	799–801 (267)	898–900 (300)	964–966 (322)	967–969 (323)	970–972 (324)	1000–1002 (334)	1021–1023 (341)	1108–1110 (370)	1276–1278 (426)	1318–1320 (440)	1339–1341 (447)	1663–1665 (555)
Reference strain														
CPV-2														
M38245 (USA)	GCA (Ala)	ATG (Met)	TTT (Phe)	GCT (Ala)	ACA (Thr)	AAC (Asn)	TAT (Tyr)	GCT (Ala)	CCA (Pro)	CAA (Gln)	AAT (Asn)	ACA (Thr)	ATA (Ile)	GTA (Val)
CPV-2a														
M24000 (USA)	GCA (Ala)	TTG (Leu)	TTT (Phe)	GGT (Gly)	ACA (Thr)	AAC (Asn)	TAT (Tyr)	GCT (Ala)	CCA (Pro)	CAA (Gln)	AAT (Asn)	ACA (Thr)	ATA (Ile)	ATA (Val)
FJ197847 (vaccine)	GCA (Ala)	ATG (Met)	TTT (Phe)	GGT (Gly)	ACA (Thr)	AAC (Asn)	TAT (Tyr)	GCT (Ala)	CCA (Pro)	CAA (Gln)	AAT (Asn)	ACA (Thr)	ATA (Ile)	GTA (Val)
New CPV-2a														
AY742953 (USA)	GCA (Ala)	TTG (Leu)	TTT(Phe)	GGT (Gly)	ACA (Thr)	AAC (Asn)	TAT (Tyr)	GCT (Ala)	CCA (Pro)	CAA (Gln)	AAT (Asn)	ACA (Thr)	ATA (Ile)	GTA (Val)
KF676668 (China)	GCA (Ala)	TTG (Leu)	TAT(Tyr)	GGT (Gly)	ACA (Thr)	AAC (Asn)	ATT (Ile)	GCT (Ala)	CCA (Pro)	CAA (Gln)	AAT (Asn)	GCA (Ala)	ATA (Ile)	GTA (Val)
KR611488 (China)	GCA (Ala)	TTG (Leu)	TAT(Tyr)	GGT (Gly)	ACA (Thr)	AAC (Asn)	ATT (Ile)	GCT (Ala)	CCA (Pro)	CAA (Gln)	AAT (Asn)	GCA (Ala)	ATA (Ile)	GTA (Val)
JQ686671 (China)	GCA (Ala)	TTG (Leu)	TAT(Tyr)	GGT (Gly)	ACA (Thr)	AAC (Asn)	ATT (Ile)	GCT (Ala)	CCA (Pro)	CAA (Gln)	AAT (Asn)	GCA (Ala)	ATA (Ile)	GTA (Val)
CPV-2b														
M78452 (USA)	GCA (Ala)	TTG (Leu)	TTT (Phe)	GGT (Gly)	ACA (Thr)	AAC (Asn)	TAT (Tyr)	GCT (Ala)	CCA (Pro)	CAA (Gln)	GAT (Asp)	ACA (Thr)	ATA (Ile)	GTA (Val)
New CPV-2b														
AY742955 (USA)	GCA (Ala)	TTG (Leu)	TTT(Phe)	GGT (Gly)	ACA (Thr)	AAC (Asn)	TAT (Tyr)	GCT (Ala)	CCA (Pro)	CAA (Gln)	GAT (Asp)	ACA (Thr)	ATA (Ile)	GTA (Val)
JQ268284 (China)	GCA (Ala)	TTG (Leu)	TAT(Tyr)	GGT (Gly)	ACA (Thr)	AAC (Asn)	ATT (Ile)	GCT (Ala)	CCA (Pro)	CAA (Gln)	GAT (Asp)	GCA (Ala)	ATA (Ile)	GTA (Val)
KR611459 (China)	GCA (Ala)	TTG (Leu)	TAT(Tyr)	GGT (Gly)	ACA (Thr)	AAC (Asn)	ATT (Ile)	GCT (Ala)	CCA (Pro)	CAA (Gln)	GAT (Asp)	GCA (Ala)	ATA (Ile)	GTA (Val)
KR611461 (China)	GCA (Ala)	TTG (Leu)	TAT(Tyr)	GGT (Gly)	ACA (Thr)	AAC (Asn)	ATT (Ile)	GCT (Ala)	CCA (Pro)	CAA (Gln)	GAT (Asp)	GCA (Ala)	ATA (Ile)	GTA (Val)
CPV-2c														
FJ005235 (USA)	GCA (Ala)	TTG (Leu)	TTT (Phe)	GCT (Ala)	ACA (Thr)	AAC (Asn)	TAT (Tyr)	GCT (Ala)	CCA (Pro)	CAA (Gln)	GAA (Glu)	ACA (Thr)	ATA (Ile)	GTA (Val)
FJ222821 (Italy)	GCA (Ala)	TTG (Leu)	TTT (Phe)	GGT (Gly)	ACA (Thr)	AAC (Asn)	TAT (Tyr)	GCT (Ala)	CCA (Pro)	CAA (Gln)	GAA (Glu)	ACA (Thr)	ATA (Ile)	GTA (Val)
KY937650 (China)	GGA (Gly)	TTG (Leu)	TAT(Tyr)	GGT (Gly)	ACA (Thr)	AAC (Asn)	ATT (Ile)	GCT (Ala)	CCA (Pro)	CAA (Gln)	GAA (Glu)	ACA (Thr)	ATA (Ile)	GTA (Val)
KU244254 (Taiwan)	GCA (Ala)	TTG (Leu)	TAT (Tyr)	GGT (Gly)	ACA (Thr)	AAC (Asn)	ATT (Ile)	GCT (Ala)	CCA (Pro)	CGA (Arg)	GAA (Glu)	ACA (Thr)	ATA (Ile)	GTA (Val)
AB120727 (Vietnam)	GCA (Ala)	TTG (Leu)	TTT (Phe)	GGT (Gly)	ACA (Thr)	AAC (Asn)	TAT (Tyr)	GCT (Ala)	CCG (Pro)	CAA (Gln)	GAA (Glu)	ACA (Thr)	ATA (Ile)	GTA (Val)
This study														
CPV-2a														
DN33/MK357724	GCA (Ala)	ATG (Met)	TTT (Phe)	GCT (Ala)	ACA (Thr)	AAC (Asn)	TAT (Tyr)	GCT (Ala)	CCA (Pro)	CAA (Gln)	AAT (Asn)	ACA (Thr)	ATA (Ile)	GTA (Val)
CPV-2c														
HN7AA/MK357734	GGA (Gly)	TTG (Leu)	TAT (Tyr)	GGT (Gly)	ACA (Thr)	AAC (Asn)	ATT (Ile)	GCT (Ala)	CCA (Pro)	CGA (Arg)	GAA (Glu)	ACA (Thr)	ATA (Ile)	GTA (Val)
HN4AA/MK357735	GGA (Gly)	TTG (Leu)	TAT (Tyr)	GGT (Gly)	ACA (Thr)	AAC (Asn)	ATT (Ile)	GCC (Ala)	CCA (Pro)	CGA (Arg)	GAA (Glu)	ACA (Thr)	ATG (Met)	GTA (Val)
N11AA/MK357736	GGA (Gly)	TTG (Leu)	TAT (Tyr)	GGT (Gly)	ACA (Thr)	AAC (Asn)	ATT (Ile)	GCT (Ala)	CCA (Pro)	CGA (Arg)	GAA (Glu)	ACA (Thr)	ATG (Met)	GTA (Val)
HN37AA/K357737	GGA (Gly)	TTG (Leu)	TAT (Tyr)	GGT (Gly)	ACA (Thr)	AAC (Asn)	ATT (Ile)	GCT (Ala)	CCA (Pro)	CGA (Arg)	GAA (Glu)	ACA (Thr)	ATG (Met)	GTA (Val)
HCM4/MK357725	GGA (Gly)	TTG (Leu)	TAT (Tyr)	GGT (Gly)	ACA (Thr)	AAC (Asn)	ATT (Ile)	GCT (Ala)	CCA (Pro)	CGA (Arg)	GAA (Glu)	ACA (Thr)	ATG (Met)	GTA (Val)
HCM16/MK357726	GGA (Gly)	TTG (Leu)	TAT (Tyr)	GGT (Gly)	ACA (Thr)	AAC (Asn)	ATT (Ile)	GCT (Ala)	CCA (Pro)	CGA (Arg)	GAA (Glu)	ACA (Thr)	ATG (Met)	GTA (Val)
HCM22/MK357727	GGA (Gly)	TTG (Leu)	TAT (Tyr)	GGT (Gly)	ACA (Thr)	AAC (Asn)	ATT (Ile)	GCT (Ala)	CCA (Pro)	CGA (Arg)	GAA (Glu)	ACA (Thr)	ATA (Ile)	GTA (Val)
HCM25/MK357728	GGA (Gly)	TTG (Leu)	TAT (Tyr)	GGT (Gly)	ACA (Thr)	AAC (Asn)	ATT (Ile)	GCT (Ala)	CCA (Pro)	CGA (Arg)	GAA (Glu)	ACA (Thr)	ATG (Met)	GTA (Val)
HCM27/MK357729	GGA (Gly)	TTG (Leu)	TAT (Tyr)	GGT (Gly)	ACA (Thr)	AAC (Asn)	ATT (Ile)	GCT (Ala)	CCA (Pro)	CGA (Arg)	GAA (Glu)	ACA (Thr)	ATG (Met)	GTA (Val)
HCM32/MK357730	GGA (Gly)	TTG (Leu)	TAT (Tyr)	GGT (Gly)	ACA (Thr)	AAC (Asn)	ATT (Ile)	GCT (Ala)	CCA (Pro)	CGA (Arg)	GAA (Glu)	ACA (Thr)	ATG (Met)	GTA (Val)
HCM48/MK357731	GGA (Gly)	TTG (Leu)	TAT (Tyr)	GGT (Gly)	ACA (Thr)	AAC (Asn)	ATT (Ile)	GCT (Ala)	CCA (Pro)	CGA (Arg)	GAA (Glu)	ACA (Thr)	ATG (Met)	GTA (Val)
HCM50/MK357732	GGA (Gly)	TTG (Leu)	TAT (Tyr)	GGT (Gly)	ACA (Thr)	AAC (Asn)	ATT (Ile)	GCT (Ala)	CCA (Pro)	CGA (Arg)	GAA (Glu)	ACA (Thr)	ATG (Met)	GTA (Val)
DN8/MK357721	GGA (Gly)	TTG (Leu)	TAT (Tyr)	GGT (Gly)	ACA (Thr)	AAC (Asn)	ATT (Ile)	GCT (Ala)	CCA (Pro)	CGA (Arg)	GAA (Glu)	ACA (Thr)	ATG (Met)	GTA (Val)
DN18/MK357722	GGA (Gly)	TTG (Leu)	TAT (Tyr)	GGT (Gly)	ACA (Thr)	AAC (Asn)	ATT (Ile)	GCT (Ala)	CCA (Pro)	CGA (Arg)	GAA (Glu)	ACA (Thr)	ATG (Met)	GTA (Val)
DN26/MK357723	GGA (Gly)	TTG (Leu)	TAT (Tyr)	GGT (Gly)	ACA (Thr)	AAC (Asn)	ATT (Ile)	GCT (Ala)	CCA (Pro)	CGA (Arg)	GAA (Glu)	ACA (Thr)	ATG (Met)	GTA (Val)

## Discussion

CPV-2c is currently the most prevalent genotype in Asian countries [17–22]. The first Asian CPV-2c case was detected in Vietnam in 2004 [16]. However, information regarding the current genotype prevalence of CPV-2 strains in Vietnam is limited. This study is the first to investigate the genotype prevalence of CPV-2 in 3 regions of Vietnam. Similar to other Asian countries [17–22], the CPV-2c variant appears to be the most prevalent genotype in the dog population in all regions of Vietnam. Surprisingly, no CPV-2b cases were found in Vietnam in the present study; however, according to previous reports, CPV-2b had the highest detection rate in Vietnam in 2004 [16] (Table 3). The reasons for this discrepancy may be as follows: i) current CPV-2b vaccines are effective in preventing and controlling the disease in Vietnam; or ii) there is limited or no protection from current vaccines against the CPV-2c variant, and this variant rapidly replaced the previous circulation of CPV-2 strains. As such, we used a SYBR Green-based real-time PCR assay, which was sensitive, specific and reliable for the detection of CPV-2, FPV and porcine parvovirus DNA [25]. However, there were three parvovirus-positive cases from northern Vietnam for which differentiation was not possible by our SimpleProbe® real-time PCR assay. This issue may be due to i) unusual changes in the probe sequence region [26]; or ii) the infection of Vietnamese dogs by other parvovirus species for which the sequence analysis has not been investigated.

**Table 3:** Review of CPV-2 genotyping in Vietnam

Study period	Region of Vietnam	Genotype of CPV-2			References
		2a	2b	2c	
2003–2004	Northern and Southern	0	8	1	Nakamura et al., 2004 [16]
2016–2018	Northern, Central and Southern	6	0	251	This study

The country of Vietnam is divided into three regions with different climates. Northern Vietnam has a humid subtropical climate, while the central region has a tropical monsoon climate, and southern Vietnam has a tropical savanna climate. Another factor that may affect the study is the humidity in Vietnam, which is 84–100% on average. However, because of differences in latitude and topography, the climate tends to be markedly different across regions, which may cause the difference in the timing of parvovirus outbreaks in specific regions. In the northern part of the country, due to more seasons than other regions (4 seasons in total), the seasonal periods of parvovirus outbreaks are usually from October to November (winter transition time), from December to January (spring transition time), and from March to May, with a peak in April (summer transition time) (Fig. 1). Northern areas show a disease period spread over many months, whereas outbreaks often occur at a more concrete, specific time in the central and southern parts of the country. In fact, in central Vietnam, the period from December to March is the time when parvovirus infection increased rapidly in dogs. In the southern part of the country, small dogs are more likely to be affected during the period from June to August when the weather is beginning to switch from the dry to rainy season. Thus, CPV-2 disease can occur in Vietnam at any time of the year depending on the geographic location; however, when there is a change in climate, temperature and humidity make puppies susceptible to infection and cause CPV-2 to become widespread in the environment due to prolonged survival time. Various contradicting studies emphasize the disease’s seasonality in different specific geographical regions. Some scientists have indicated that the highest incidence of this disease is detected in spring and summer [28, 29], while the opposite is true for other locations [30–33].

Dogs aged 3 months to 6 months accounted for the highest detection rate (129/260; 49.62%), while this rate for the 1 month to 2 month age group was 26.92% (70/260), and the rate for dogs over 6 months old was only 8.85% (23/260). For 38 samples, the age group was not reported. The percentage of dogs under 6 months of age who were greatly affected has been previously reported [29, 34]. The reason dogs from 3 months to 6 months old are easily susceptible to CPV is due to the decrease in maternal antibodies [35, 36]. In addition, the higher incidence of CPV-2 in dogs younger than 6 months old might be due to quicker intestinal crypt cell multiplication with a higher mitotic index by changes in bacterial flora and daily diet during weaning time [1, 36]. Very few cases are recorded over 1 year of age; the cause may be slight exposure to the virus, leading to antibody production in the host; previous vaccination of animals; or another reason not yet identified. Dogs in the 7 to 12 month age group still exhibit CPV disease, which may be because of poor vaccine preservation or incorrect vaccination timing [37].

We found that the proportion of infected females was less than that of males. Of 260 positive cases, 94 were females (36.15%), and 133 (51.15%) were males. Coincidently, the current finding is consistent with studies by Thomas et al. [38] and Gombac et al. [39], as well as other reliable publications [34, 38, 40–42]. Many assumptions could be analyzed; however, the most relevant cause could be that the majority of samples were collections from male dogs during the study. Another explanation for this result could be that male dogs are more likely to be infected; in fact, more male dogs were admitted for treatment than female dogs. However, there is reportedly no influence of sex on the incidence of CPV [43, 44]. The high incidence in male dogs may also be due to some behaviors and habits of pet owners or the hobby of selecting and keeping male dogs [45].

A large number of dogs have been vaccinated against CPV-2 but still suffer from the disease, with most of the cases (32/44; 72.72%) 3 months of age and older. This issue may be related to failure to properly use the vaccine or to preserve the vaccine [11, 46]. Many studies have shown that CPV-2 and CPV-2b vaccines provide protection against the CPV-2a/2b virus and provide complete protection against the new CPV-2c variant in dogs for up to 9 years [47–49]. However, there are some disagreements regarding the use of traditional vaccines against the new antigen variant CPV-2c [50, 51]. Similar to a study conducted in Taiwan, four of 22 CPV-2c-infected dogs died despite vaccination, including one adult dog that completed the vaccination program [17]. This phenomenon has also been seen in Vietnam where 100% of vaccinated positive cases were of genotype 2c. Many CPV-2 vaccines are being used in Vietnam; however, there is little documentation of the effects of CPV-2 vaccination against the current CPV-2c variant. Therefore, the efficacy of the current vaccine against the CPV-2c variant remains to be evaluated.

Currently, there are many publications regarding the circulation of the CPV-2c strain in many countries in Asia [17–21, 23–25, 52, 53] Although the first publication on CPV-2c was in Vietnam in 2004 [16], the CPV-2c variant has not been prevalent in Asia. Surprisingly, novel Asian CPV-2c isolates were reported in China [18–21], Taiwan [17, 22], Laos [23], and Thailand [24]. This finding suggests that novel CPV-2c is now becoming increasingly prevalent in Asian countries. Based on the results of the phylogenetic analysis, the cause for the large amount of CPV-2c detected in Vietnam is the invasion of a foreign strain from 2016 to 2018. In addition, there was no phylogenetical relation between Vietnamese CPV-2c and prototype CPV-2c strains (European or American strains) or the past Vietnamese CPV-2c in 2004, while these strains are similar to the recent Asian CPV-2c strains (Fig. 4). Phylogenetic analysis revealed that the recent Vietnamese CPV-2c strains share a common evolutionary origin with Asian CPV-2c strains. This finding could be explained by dog imports from China and other neighboring nations with a lack of oversight due to a long shared border with these countries and the effort needed from authorities to apply restrictions.

Amino acid substitutions of Ala5Gly, Phe267Tyr, Tyr324Ile and Gln370Arg of CPV-2c have been observed in China [18, 20, 21] and Taiwan [17, 22]. However, the functions of CPV-2 residues 5, 267, 324 and 370 are still unknown and remain to be elucidated. The substitution of Ile447Met is unique to the Vietnamese CPV-2c strains. Residue 447 is not exposed on the capsid surface [54], and substitutions in this position may not affect the antigenicity of the virus. However, Agbandie’s study showed that the binding of DNA within the internal surface of the parvovirus protein shell may cause specific conformational changes in the protein [54]. Therefore, the function of residue 447 remains to be elucidated.

## Conclusions

This study is the first CPV-2 epidemiological survey in Vietnam to document the presence and relative distribution of CPV-2 variants in three regions of Vietnam over the past few years. Phylogenetic analysis demonstrated that the recent Vietnamese CPV-2c isolates share a common evolutionary origin with Asian CPV-2c strains. It is important for veterinarians and dog owners to understand the current geno-prevalence of CPV-2 and effective prevention methods based on outbreak periods.

## Additional file

Additional file 1: The genotypes of 260 canine parvovirus type 2 isolates collected from Vietnamese dogs. (DOCX 76 kb)

## Abbreviations

CPV-2: Canine parvovirus type 2
FPV: Feline panleukopenia virus
PCR: Polymerase chain reaction
