# Supplementary material for: Molecular epidemiology of canine parvovirus type 2 in Vietnam from November 2016 to February 2018
Source: Virol J. 2019 Apr 27;16:52. doi: 10.1186/s12985-019-1159-z (PMC6486976; doi:10.1186/s12985-019-1159-z)
Supplement: Supplementary file 1 — The genotypes of 260 canine parvovirus type 2 isolates collected from Vietnamese dogs. (DOCX 76 kb) [file 12985_2019_1159_MOESM1_ESM.docx]

Additional file 1 The genotypes of 260 canine parvovirus type 2 isolates collected from Vietnamese dogs

| Collection date | Strain | Genotype | Melting temperature | Cq | Region | Age | Sex | Vaccination | Death |
| --- | --- | --- | --- | --- | --- | --- | --- | --- | --- |
| 8.3.2017 | HCM-1 | CPV-2c | 52.6 | 16.045 | South | N/A | M | Yes | N/A |
| 8.3.2017 | HCM-2 | CPV-2c | 52.4 | 13.808 | South | 4 M | F | No | N/A |
| 17.3.2017 | HCM-3 | CPV-2c | 52.8 | 14.779 | South | 3 M | M | N/A | No |
| 17.3.2017 | HCM-4 | CPV-2c | 52.9 | 7.147 | South | 4 M | N/A | N/A | No |
| 17.3.2017 | HCM-5 | CPV-2c | 52.9 | 17.893 | South | 3 M | M | N/A | N/A |
| 20.3.2017 | HCM-6 | CPV-2c | 52.2 | 27.457 | South | 2 M | F | N/A | N/A |
| 20.3.2017 | HCM-8 | CPV-2c | 52.6 | 26.457 | South | N/A | M | N/A | N/A |
| 22.3.2017 | HCM-9 | CPV-2c | 52.3 | 14.71 | South | 3 M | F | Yes | No |
| 25.3.2017 | HCM-10 | CPV-2c | 52.8 | 11.101 | South | 2 M | N/A | N/A | No |
| 14.4.2017 | HCM-11 | CPV-2c | 52.8 | 11.6 | South | 1 Y | M | N/A | No |
| 28.4.2017 | HCM-12 | CPV-2c | 52.9 | 19.8 | South | N/A | N/A | N/A | N/A |
| 2.5.2017 | HCM-14 | CPV-2c | 53 | 11.2 | South | 5 M | M | N/A | N/A |
| 2.5.2017 | HCM-15 | CPV-2c | 52.9 | 12.6 | South | 2 M | M | Yes | Yes |
| 9.5.2017 | HCM-16 | CPV-2c | 52.8 | 17.4 | South | 6 M | F | No | No |
| 15.5.2017 | HCM-17 | CPV-2c | 52.8 | 15.2 | South | 4 M | M | N/A | No |
| 2.6.2017 | HCM-18 | CPV-2c | 52.4 | 13.03 | South | 2 M | N/A | No | N/A |
| 2.6.2017 | HCM-19 | CPV-2c | 52.2 | 13.1 | South | N/A | N/A | N/A | N/A |
| 2.6.2017 | HCM-20 | CPV-2c | 52.2 | 12.1 | South | 5 M | F | N/A | N/A |
| 3.6.2017 | HCM-21 | CPV-2a | 50.2 | 11.7 | South | 3 M | M | N/A | N/A |
| 3.6.2017 | HCM-22 | CPV-2c | 52.6 | 7.2 | South | 6 M | F | N/A | N/A |
| 7.6.2017 | HCM-23 | CPV-2c | 52.9 | 26.01 | South | 4 M | M | N/A | N/A |
| 7.6.2017 | HCM-24 | CPV-2c | 53.1 | 17.9 | South | 2 M | M | Yes | Yes |
| 11.6.2017 | HCM-25 | CPV-2c | 52.4 | 7.8 | South | 4 M | N/A | N/A | N/A |
| 18.6.2017 | HCM-26 | CPV-2c | 52.2 | 20.87 | South | 4 M | M | No | No |
| 20.6.2017 | HCM-27 | CPV-2c | 52.6 | 12.9 | South | 4 M | F | No | Yes |
| 20.6.2017 | HCM-28 | CPV-2c | 52.8 | 12.1 | South | 5 M | M | Yes | No |
| 20.6.2017 | HCM-29 | CPV-2c | 52.9 | 17.8 | South | 2 M | M | No | Yes |
| 27.6.2017 | HCM-30 | CPV-2c | 52.9 | 8.2 | South | 3 M | M | N/A | Yes |
| 30.6.2017 | HCM-31 | CPV-2c | 52.3 | 11.4 | South | N/A | M | No | No |
| 18.7.2017 | HCM-32 | CPV-2c | 53.2 | 10.5 | South | N/A | F | No | No |
| 18.7.2017 | HCM-33 | CPV-2c | 53.1 | 22.3 | South | 7 M | F | Yes | No |
| 18.7.2017 | HCM-34 | CPV-2c | 53.1 | 25.3 | South | 2 M | F | N/A | N/A |
| 18.7.2017 | HCM-35 | CPV-2c | 52.4 | 8.8 | South | 1 M | M | N/A | N/A |
| 19.7.2017 | HCM-36 | CPV-2c | 52.7 | 14.1 | South | 7 M | M | No | Yes |
| 19.7.2017 | HCM-37 | CPV-2c | 52.2 | 18.8 | South | 6 M | M | N/A | N/A |
| 19.7.2017 | HCM-38 | CPV-2c | 52.3 | 31.2 | South | 11 M | F | No | No |
| 19.7.2017 | HCM-39 | CPV-2c | 53 | 12.8 | South | 3 M | M | Yes | Yes |
| 19.7.2017 | HCM-40 | CPV-2c | 52.8 | 11.7 | South | 4 M | F | Yes | No |
| 20.7.2017 | HCM-41 | CPV-2c | 52.8 | 14.05 | South | N/A | F | N/A | N/A |
| 20.7.2017 | HCM-42 | CPV-2c | 52.8 | 19.01 | South | 4 M | M | No | N/A |
| 20.7.2017 | HCM-43 | CPV-2c | 52.5 | 23.76 | South | 4 M | N/A | N/A | No |
| 20.7.2017 | HCM-44 | CPV-2c | 52.9 | 17.8 | South | 6 M | N/A | N/A | N/A |
| 20.7.2017 | HCM-45 | CPV-2c | 52.8 | 13.05 | South | 2 M | M | No | N/A |
| 20.7.2017 | HCM-46 | CPV-2c | 52.9 | 14.1 | South | 2 M | F | No | N/A |
| 21.7.2017 | HCM-47 | CPV-2c | 52.8 | 18.7 | South | N/A | N/A | N/A | N/A |
| 21.7.2017 | HCM-48 | CPV-2c | 52.9 | 14.02 | South | 5 M | M | Yes | No |
| 21.7.2017 | HCM-49 | CPV-2c | 52.9 | 15.6 | South | 6 M | F | N/A | N/A |
| 21.7.2017 | HCM-50 | CPV-2c | 52.7 | 12.2 | South | 4 M | M | No | No |
| 22.7.2017 | HCM-51 | CPV-2c | 52.8 | 11.5 | South | 9 M | F | Yes | No |
| 22.7.2017 | HCM-52 | CPV-2c | 52.8 | 13.7 | South | 2 M | M | No | No |
| 22.7.2017 | HCM-53 | CPV-2c | 52.9 | 11.3 | South | 3 M | F | No | Yes |
| 22.7.2017 | HCM-54 | CPV-2c | 52.8 | 17.6 | South | 7 M | M | Yes | No |
| 22.7.2017 | HCM-55 | CPV-2c | 52.9 | 14.7 | South | 3 M | F | Yes | Yes |
| 23.7.2017 | HCM-56 | CPV-2c | 52.9 | 11.3 | South | 4 M | M | N/A | Yes |
| 23.7.2017 | HCM-57 | CPV-2c | 52.8 | 15.1 | South | N/A | F | N/A | N/A |
| 23.7.2017 | HCM-58 | CPV-2c | 52.6 | 14.2 | South | 4 M | N/A | N/A | N/A |
| 23.7.2017 | HCM-59 | CPV-2c | 52.8 | 16.8 | South | 10 M | M | No | No |
| 23.7.2017 | HCM-60 | CPV-2c | 52.2 | 10.7 | South | 6 M | F | No | Yes |
| 20.9.2017 | HCM-61 | CPV-2c | 53.1 | 20.4 | South | N/A | M | N/A | N/A |
| 24.9.2017 | HCM-62 | CPV-2c | 52.3 | 17.6 | South | 4 M | N/A | N/A | N/A |
| 24.9.2017 | HCM-63 | CPV-2a | 50.3 | 14.3 | South | 5 M | F | N/A | N/A |
| 24.9.2017 | HCM-64 | CPV-2c | 52.8 | 16.6 | South | N/A | M | No | N/A |
| 1.10.2017 | HCM-65 | CPV-2c | 52.2 | 12.1 | South | 4 M | F | Yes | N/A |
| 12.10.2017 | HCM-66 | CPV-2c | 52.4 | 17.3 | South | 2 M | M | No | Yes |
| 21.10.2017 | HCM-67 | CPV-2c | 52.8 | 13.4 | South | 3 M | M | Yes | Yes |
| 29.10.2017 | HCM-68 | CPV-2c | 52.6 | 12.2 | South | N/A | F | Yes | No |
| 5.11.2016 | DN-1 | CPV-2c | 52.2 | 34.6 | Central | 2 M | M | N/A | N/A |
| 10.11.2016 | DN-2 | CPV-2c | 52.9 | 17.08 | Central | 4 M | M | N/A | N/A |
| 10.11.2016 | DN-3 | CPV-2c | 53 | 9.8 | Central | 2 M | M | N/A | N/A |
| 10.11.2016 | DN-4 | CPV-2c | 52.5 | 27.1 | Central | 3 M | N/A | N/A | N/A |
| 17.11.2016 | DN-5 | CPV-2c | 52.5 | 15.5 | Central | N/A | F | N/A | N/A |
| 3.12.2016 | DN-6 | CPV-2c | 52.5 | 15.1 | Central | 3 M | M | N/A | N/A |
| 3.12.2016 | DN-7 | CPV-2c | 52.4 | 19.2 | Central | 2 M | M | N/A | N/A |
| 3.12.2016 | DN-8 | CPV-2c | 52.5 | 14.1 | Central | N/A | M | Yes | N/A |
| 3.12.2016 | DN-9 | CPV-2c | 53 | 28.2 | Central | N/A | M | Yes | No |
| 3.12.2016 | DN-10 | CPV-2c | 52.2 | 13.8 | Central | N/A | F | No | Yes |
| 5.12.2016 | DN-11 | CPV-2c | 52.8 | 17.008 | Central | 4 M | M | N/A | N/A |
| 7.12.2016 | DN-12 | CPV-2c | 53 | 10.2 | Central | 3 M | M | N/A | No |
| 8.12.2016 | DN-13 | CPV-2c | 52.9 | 17.5 | Central | 4 M | F | N/A | N/A |
| 15.12.2016 | DN-14 | CPV-2c | 52.8 | 8.1 | Central | 4 M | M | N/A | N/A |
| 15.12.2016 | DN-15 | CPV-2c | 52.9 | 13.8 | Central | 2 M | F | N/A | N/A |
| 15.12.2016 | DN-16 | CPV-2c | 53.1 | 16.4 | Central | 5 M | N/A | N/A | N/A |
| 17.12.2016 | DN-18 | CPV-2c | 52.7 | 12.3 | Central | 2 M | M | N/A | No |
| 18.12.2016 | DN-19 | CPV-2c | 52.8 | 8.5 | Central | N/A | M | N/A | No |
| 21.12.2016 | DN-20 | CPV-2c | 52.9 | 15.04 | Central | N/A | N/A | N/A | N/A |
| 21.12.2016 | DN-21 | CPV-2c | 52.7 | 17.3 | Central | N/A | N/A | N/A | N/A |
| 21.12.2016 | DN-22 | CPV-2c | 52.8 | 16.7 | Central | N/A | F | N/A | No |
| 21.12.2016 | DN-23 | CPV-2c | 52.5 | 17.3 | Central | 6 M | F | N/A | Yes |
| 21.12.2016 | DN-24 | CPV-2c | 53.1 | 13.8 | Central | 2 M | M | N/A | Yes |
| 21.12.2016 | DN-25 | CPV-2c | 52.3 | 27.5 | Central | 2 M | F | N/A | N/A |
| 22.12.2016 | DN-26 | CPV-2c | 52.7 | 8.8 | Central | 3 M | M | N/A | N/A |
| 26.12.2016 | DN-29 | CPV-2c | 53 | 16.2 | Central | 4 M | F | N/A | N/A |
| 26.12.2016 | DN-30 | CPV-2c | 52.9 | 17.6 | Central | 6 M | M | N/A | N/A |
| 26.12.2016 | DN-31 | CPV-2c | 52.9 | 18.2 | Central | 2 M | M | N/A | N/A |
| 29.12.2016 | DN-32 | CPV-2c | 52.7 | 14.9 | Central | 4 M | F | N/A | N/A |
| 2.1.2017 | DN-33 | CPV-2a | 50.1 | 23.6 | Central | 2 M | M | No | N/A |
| 4.1.2017 | DN-34 | CPV-2c | 53.1 | 12.15 | Central | 7 M | F | No | N/A |
| 6.1.2017 | DN-35 | CPV-2c | 52.8 | 17.4 | Central | 2 M | F | Yes | Yes |
| 6.1.2017 | DN-37 | CPV-2c | 52.9 | 18.4 | Central | N/A | F | N/A | N/A |
| 14.1.2017 | DN-38 | CPV-2c | 53.2 | 27.1 | Central | 2 M | M | N/A | N/A |
| 14.1.2017 | DN-39 | CPV-2c | 53.2 | 14.05 | Central | 2 M | M | Yes | N/A |
| 19.1.2017 | DN-40 | CPV-2c | 53.2 | 19.2 | Central | 4 M | F | No | No |
| 19.1.2017 | DN-43 | CPV-2c | 53.1 | 13.4 | Central | 3 M | M | No | Yes |
| 19.1.207 | DN-44 | CPV-2c | 53.1 | 17.03 | Central | 6 M | M | No | Yes |
| 23.1.2017 | DN-45 | CPV-2c | 53.2 | 15.51 | Central | N /A | M | N/A | No |
| 23.1.2017 | DN-46 | CPV-2c | 53.1 | 8.1 | Central | 2 M | N/A | N/A | Yes |
| 5.2.2017 | DN-47 | CPV-2c | 53 | 13.1 | Central | 3 M | N/A | N/A | Yes |
| 8.2.2017 | DN-48 | CPV-2c | 52.8 | 19.07 | Central | N/A | N/A | N/A | N/A |
| 9.2.2017 | DN-49 | CPV-2c | 53.1 | 13.7 | Central | 3 M | M | N/A | Yes |
| 14.2.2017 | DN-51 | CPV-2c | 53.1 | 12.08 | Central | 4,5 M | F | No | No |
| 14.2.2017 | DN-52 | CPV-2c | 53 | 12.74 | Central | 2 M | M | Yes | No |
| 15.2.2017 | DN-53 | CPV-2c | 53.1 | 17.6 | Central | 4 M | M | No | No |
| 20.2.2017 | DN-54 | CPV-2c | 53.1 | 13.6 | Central | 3 M | N/A | N/A | Yes |
| 20.2.2017 | DN-55 | CPV-2c | 53 | 20 | Central | 8 M | N/A | N/A | Yes |
| 20.2.2017 | DN-56 | CPV-2c | 52.9 | 11.7 | Central | 1 Y | N/A | N/A | No |
| 20.2.2017 | DN-57 | CPV-2c | 53.2 | 11.6 | Central | 3 M | F | No | N/A |
| 20.2.2017 | DN-58 | CPV-2c | 53.1 | 18.3 | Central | 11 M | M | Yes | N/A |
| 21.2.2017 | DN-59 | CPV-2c | 53.2 | 21.3 | Central | 2 M | M | No | N/A |
| 21.2.2017 | DN-60 | CPV-2c | 53.1 | 17.4 | Central | N/A | F | N/A | Yes |
| 21.2.2017 | DN-61 | CPV-2c | 53.1 | 12.7 | Central | 4 M | M | Yes | No |
| 24.2.2017 | DN-62 | CPV-2c | 52.9 | 16.5 | Central | 7 M | M | No | No |
| 24.2.2017 | DN-63 | CPV-2c | 53 | 13.3 | Central | N/A | F | No | Yes |
| 24.2.2017 | DN-64 | CPV-2c | 53.2 | 20.01 | Central | 4 M | M | N/A | No |
| 24.2.2017 | DN-65 | CPV-2c | 52.6 | 17.9 | Central | 3 M | M | Yes | No |
| 24.2.2017 | DN-66 | CPV-2c | 52.8 | 19.9 | Central | 5 M | M | No | No |
| 25.2.2017 | DN-67 | CPV-2c | 52.8 | 13.5 | Central | 2 M | F | No | Yes |
| 26.2.2017 | DN-68 | CPV-2c | 52.9 | 14.8 | Central | 1.5 M | F | No | No |
| 26.2.2017 | DN-69 | CPV-2c | 52.2 | 14.1 | Central | 2 M | M | No | No |
| 1.3.2017 | DN-70 | CPV-2c | 52.4 | 18.4 | Central | N/A | M | N/A | N/A |
| 1.3.2017 | DN-71 | CPV-2c | 52.3 | 20.02 | Central | 6 M | F | No | N/A |
| 1.3.2017 | DN-72 | CPV-2c | 52.2 | 20.73 | Central | 2 M | M | Yes | Yes |
| 1.3.2017 | DN-73 | CPV-2c | 52.2 | 29.08 | Central | 4 M | F | Yes | No |
| 3.3.2017 | DN-74 | CPV-2c | 52.8 | 15.5 | Central | N/A | M | No | No |
| 3.3.2017 | DN-75 | CPV-2c | 53.1 | 13.1 | Central | 3 M | N/A | N/A | N/A |
| 4.3.2017 | DN-76 | CPV-2c | 52.6 | 17.1 | Central | 4 M | N/A | N/A | N/A |
| 5.3.2017 | DN-77 | CPV-2c | 52.9 | 15.06 | Central | 3 M | N/A | N/A | N/A |
| 5.3.2017 | DN-79 | CPV-2c | 52.8 | 15.5 | Central | 3 M | M | N/A | No |
| 5.3.2017 | DN-80 | CPV-2c | 52.9 | 20.1 | Central | 5 M | F | Yes | No |
| 8.3.2017 | DN-81 | CPV-2c | 52.9 | 17.02 | Central | 2 M | F | N/A | N/A |
| 8.3.2017 | DN-82 | CPV-2c | 52.8 | 17.04 | Central | 2 M | M | No | N/A |
| 8.3.2017 | DN-83 | CPV-2c | 53 | 17.3 | Central | 2 M | N/A | N/A | N/A |
| 8.3.2017 | DN-84 | CPV-2c | 52.3 | 20.99 | Central | 1 M | M | No | Yes |
| 10.3.2017 | DN-86 | CPV-2c | 52.7 | 15.2 | Central | 5 M | M | N/A | No |
| 15.3.2017 | DN-87 | CPV-2c | 52.4 | 20.5 | Central | 4 M | F | Yes | No |
| 26.3.2017 | DN-88 | CPV-2c | 52.9 | 12.9 | Central | 2 M | M | N/A | No |
| 12.4.2017 | DN-89 | CPV-2c | 52.9 | 13.7 | Central | 2 M | M | No | N/A |
| 26.4.2017 | DN-90 | CPV-2c | 52.8 | 11.1 | Central | 6 M | M | N/A | N/A |
| 25.5.2017 | DN-91 | CPV-2c | 52.7 | 14.1 | Central | 8 M | M | No | N/A |
| 26.5.2017 | DN-92 | CPV-2c | 52.9 | 12.5 | Central | 3 M | M | N/A | N/A |
| 25.6.2017 | DN-93 | CPV-2c | 52.4 | 27.05 | Central | 2 M | M | N/A | N/A |
| 28.6.2017 | DN-94 | CPV-2c | 52.8 | 13.03 | Central | 11 M | F | No | N/A |
| 26.7.2017 | DN-95 | CPV-2c | 52.8 | 16.7 | Central | 4 M | F | Yes | N/A |
| 26.7.2017 | DN-97 | CPV-2c | 53.1 | 14.7 | Central | 2 M | M | N/A | N/A |
| 27.7.2017 | DN-98 | CPV-2c | 53.1 | 13.86 | Central | 2 M | M | No | Yes |
| 27.7.2017 | DN-99 | CPV-2c | 53.2 | 17.3 | Central | 2 M | M | N/A | Yes |
| 27.7.2017 | DN-100 | CPV-2c | 52.5 | 29.79 | Central | 5 M | M | N/A | No |
| 12.10.2016 | HN-1 | CPV-2c | 52.8 | 9.214 | North | 3 M | M | No | N/A |
| 12.10.2016 | HN-2 | CPV-2c | 52.9 | 12.501 | North | 3 M | F | No | N/A |
| 12.10.2016 | HN-3 | CPV-2c | 52.9 | 15.002 | North | 3 M | F | N/A | No |
| 13.10.2016 | HN-4a | CPV-2c | 52.8 | 12.027 | North | N/A | F | N/A | N/A |
| 13.10.2016 | HN-4b | CPV-2c | 52.9 | 14.452 | North | 4 M | F | N/A | N/A |
| 16.10.2016 | HN-5 | CPV-2c | 52.8 | 10.6 | North | 4 M | M | No | N/A |
| 25.10.2016 | HN-6 | CPV-2c | 52.8 | 8.8 | North | 2 M | F | No | Yes |
| 28.10.2016 | HN-28 | CPV-2c | 52.6 | 15.9 | North | 2 M | M | No | Yes |
| 28.10.2016 | HN-32 | CPV-2c | 52.9 | 17.2 | North | 6 M | F | No | No |
| 28.10.2016 | HN-33 | CPV-2c | 52.7 | 13.9 | North | 3 M | F | N/A | No |
| 29.10..2016 | HN-35 | CPV-2c | 52.3 | 18.7 | North | 11 M | F | Yes | No |
| 29.10.2016 | HN-36 | CPV-2c | 52.2 | 16.1 | North | 2 M | M | No | Yes |
| 29.10.2016 | HN-37 | CPV-2c | 52.8 | 12.2 | North | 4 M | M | Yes | No |
| 29.10.2016 | HN-38 | CPV-2c | 53 | 16.03 | North | 3 M | N/A | N/A | N/A |
| 29.10.2016 | HN-39 | CPV-2c | 53.2 | 16.6 | North | 8 M | M | Yes | No |
| 30.10.2016 | HN-40 | CPV-2c | 53.1 | 10.8 | North | 2 M | M | No | Yes |
| 30.10.2016 | HN-42 | CPV-2c | 52.7 | 12.1 | North | 4 M | F | No | N/A |
| 30.10.2016 | HN-43 | CPV-2c | 52.8 | 13.7 | North | 4 M | M | Yes | No |
| 30.10.2016 | HN-44 | CPV-2c | 52.8 | 11.232 | North | 2 M | F | No | N/A |
| 1.11.2016 | HN-45 | CPV-2c | 52.6 | 11.625 | North | 3 M | F | N/A | N/A |
| 1.11.2016 | HN-47 | CPV-2c | 52.9 | 16.766 | North | 2.5 M | F | No | N/A |
| 1.11.2016 | HN-48 | CPV-2c | 52.2 | 20.574 | North | 2 M | M | No | N/A |
| 2.11.2016 | HN-68 | CPV-2c | 52.7 | 13.783 | North | 6 M | N/A | N/A | N/A |
| 2.11.2016 | HN-69 | CPV-2c | 52.6 | 16.505 | North | 2 M | M | Yes | N/A |
| 2.11.2016 | HN-70 | CPV-2c | 52.9 | 9.478 | North | N/A | N/A | N/A | N/A |
| 2.11.2016 | HN-71 | CPV-2c | 52.8 | 11.1 | North | 9 M | F | N/A | N/A |
| 2.11.2016 | HN-72 | CPV-2c | 52.9 | 14.4 | North | 2 M | F | N/A | N/A |
| 4.11.2016 | HN-73 | CPV-2c | 52.9 | 10.6 | North | 2 M | M | N/A | N/A |
| 4.11.2016 | HN-74a | CPV-2c | 52.6 | 27.4 | North | 10 M | M | No | N/A |
| 4.11.2016 | HN-74b | CPV-2c | 52.8 | 9.7 | North | 4 M | M | No | N/A |
| 6.11.2016 | HN-75 | CPV-2c | 52.8 | 11.8 | North | 3 M | F | Yes | N/A |
| 7.11.2016 | HN-76 | CPV-2c | 52.9 | 12.9 | North | 2 M | F | No | N/A |
| 7.11.2016 | HN-77 | CPV-2c | 52.6 | 17.5 | North | 3 M | F | Yes | N/A |
| 7.11.2016 | HN-78 | CPV-2c | 52.5 | 8.7 | North | 5 M | M | N/A | N/A |
| 7.11.2016 | HN-80 | CPV-2c | 52.8 | 11.4 | North | 2 M | M | No | N/A |
| 8.11.2016 | HN-81 | CPV-2c | 52.8 | 12.1 | North | 3 M | M | N/A | N/A |
| 8.11.2016 | HN-82 | CPV-2c | 52.6 | 16.6 | North | 3 M | M | Yes | Yes |
| 8.11.2016 | HN-84 | CPV-2c | 52.5 | 16.2 | North | 4 M | F | No | Yes |
| 10.12.2016 | HN-85 | CPV-2c | 52.9 | 9.3 | North | 5 M | M | Yes | No |
| 13.12.2016 | HN-87 | CPV-2c | 52.4 | 11.9 | North | 2 M | F | No | Yes |
| 20.12.2016 | HN-88 | CPV-2c | 52.7 | 8.6 | North | 3 M | F | Yes | No |
| 22.12.2016 | HN-90 | CPV-2c | 52.6 | 15.3 | North | 2 M | M | No | Yes |
| 3.1.2017 | HN-92 | CPV-2c | 52.8 | 13.06 | North | 6 M | M | Yes | No |
| 2.4.2017 | HN-93 | CPV-2c | 53.1 | 30.4 | North | 2 M | M | No | N/A |
| 2.4.2017 | HN-93a | CPV-2c | 52.9 | 9.224 | North | 4 M | M | N/A | N/A |
| 2.4.2017 | HN-93b | CPV-2c | 52.8 | 21.7 | North | 5 M | F | No | Yes |
| 2.4.2017 | HN-95 | CPV-2c | 52.7 | 8.03 | North | 4 M | F | No | Yes |
| 5.4.2017 | HN-96 | CPV-2c | 52.8 | 11.8 | North | 2 M | M | No | No |
| 5.4.2017 | HN-97 | CPV-2c | 52.2 | 14.1 | North | 3 M | F | Yes | No |
| 5.4.2017 | HN97b | CPV-2c | 52.3 | 14.01 | North | 4 M | M | No | No |
| 5.4.2017 | HN-98 | CPV-2c | 52.5 | 18.7 | North | 7 M | F | No | Yes |
| 6.4.2017 | HN-99 | CPV-2c | 52.8 | 12.7 | North | 2 M | M | No | No |
| 8.4.2017 | HN-100 | CPV-2c | 52.3 | 19.47 | North | 2 M | M | No | Yes |
| 8.4.2017 | HN-101 | CPV-2c | 52.6 | 17.07 | North | 2 M | F | Yes | Yes |
| 8.4.2017 | HN-102 | CPV-2c | 52.7 | 8.51 | North | 8 M | F | No | No |
| 8.4.2017 | HN-103 | CPV-2c | 53.1 | 20.9 | North | 4 M | M | Yes | No |
| 8.4.2017 | HN-104 | CPV-2c | 52.6 | 14.9 | North | 3 M | M | Yes | Yes |
| 9.4.2017 | HN-105a | CPV-2c | 53 | 36.3 | North | 6 M | M | No | No |
| 9.4.2017 | HN-105b | CPV-2c | 53.1 | 21.9 | North | 6 M | F | No | No |
| 12.4.2017 | HN-107 | CPV-2c | 52.8 | 11.9 | North | 1.5 M | F | No | Yes |
| 12.4.2017 | HN-108a | CPV-2c | 52.7 | 11.6 | North | N/ A | F | N/A | N/A |
| 12.4.2017 | HN-108b | CPV-2c | 52.9 | 7.3 | North | N/A | M | N/A | N/A |
| 12.4.2017 | HN-109 | CPV-2c | 52.3 | 19.1 | North | 2 M | M | Yes | N/A |
| 9.9.2017 | HN-18AA | CPV-2c | 52.6 | 14.5 | North | 2 M | M | N/A | N/A |
| 19.11.2017 | HN-19AA | CPV-2c | 52.2 | 17.8 | North | 6 M | F | N/A | N/A |
| 25.11.2017 | HN-39AA | Untenable | N/A | 13.9 | North | 5M | M | No | N/A |
| 21.11.2017 | HN-16AA | CPV-2c | 53.1 | 20.2 | North | 2.5 M | M | N/A | N/A |
| 21.11.2017 | HN-17AA | CPV-2c | 52.9 | 15.6 | North | 6 M | F | N/A | N/A |
| 23.12.2017 | HN-40AA | Untenable | N/A | 13.1 | North | 4M | M | N/A | N/A |
| 30.12.2017 | HN-7AA | CPV-2c | 52.8 | 12.5 | North | 6 M | M | N/A | N/A |
| 30.12.2017 | HN-41AA | Untenable | N/A | 11.8 | North | 3M | M | N/A | N/A |
| 4.1.2018 | HN- 9AA | CPV-2c | 52.8 | 13.7 | North | 6 M | M | N/A | N/A |
| 4.1.2018 | HN- 3AA | CPV-2c | 52.5 | 17.3 | North | 3 M | F | N/A | N/A |
| 4.1.2018 | HN-1AA | CPV-2a | 50.3 | 31.9 | North | 9 M | M | N/A | NA |
| 5.1.2018 | HN- 5AA | CPV-2c | 52.4 | 16.4 | North | 2.5 M | F | N/A | N/A |
| 5.1.2018 | HN-2AA | CPV-2c | 52.7 | 18.5 | North | 3 M | M | N/A | N/A |
| 6.1.2018 | HN- 15AA | CPV-2a | 50.2 | 12.8 | North | 3 M | F | N/A | N/A |
| 7.1.2018 | HN-4AA | CPV-2c | 52.9 | 19.3 | North | 6M | F | N/A | N/A |
| 7.1.2018 | HN- 8AA | CPV-2c | 52.2 | 15.8 | North | 3 M | M | N/A | N/A |
| 7.1.2018 | HN- 11AA | CPV-2c | 52.7 | 17.2 | North | 1 Y | M | N/A | N/A |
| 9.1.2018 | HN- 10AA | CPV-2c | 52.4 | 13.4 | North | 2 M | F | N/A | N/A |
| 9.1.2018 | HN-13AA | CPV-2c | 52.9 | 14.8 | North | 9 M | M | N/A | N/A |
| 9.1.2018 | HN-14AA | CPV-2c | 52.6 | 11.1 | North | 4 M | M | N/A | N/A |
| 9.1.2018 | HN- 23AA | CPV-2c | 52.8 | 16.3 | North | N/A | N/A | N/A | N/A |
| 10.1.2018 | HN-6AA | CPV-2c | 52.6 | 13.5 | North | 3 M | F | N/A | N/A |
| 11.1.2018 | HN- 12AA | CPV-2c | 52.3 | 15.4 | North | 4 M | F | N/A | N/A |
| 11.1.2018 | HN- 22AA | CPV-2c | 52.6 | 19.2 | North | N/A | N/A | N/A | N/A |
| 11.1.2018 | HN- 35 AA | CPV-2c | 52.8 | 13.5 | North | 4 M | F | N/A | N/A |
| 13.1.2018 | HN- 36AA | CPV-2a | 50.3 | 10.5 | North | 2 M | F | N/A | N/A |
| 15.1.2018 | HN-33AA | CPV-2c | 52.9 | 16.7 | North | 2 M | F | N/A | N/A |
| 15.1.2018 | HN-38AA | CPV-2c | 52.9 | 14.9 | North | 3M | M | N/A | N/A |
| 17.1.2018 | HN- 20AA | CPV-2c | 53.1 | 19.8 | North | N/A | M | N/A | N/A |
| 17.1.2018 | HN- 24AA | CPV-2c | 53.2 | 21.7 | North | 5 M | M | N/A | N/A |
| 18.1.2018 | HN- 25AA | CPV-2c | 53.2 | 18.9 | North | N/A | N/A | N/A | N/A |
| 19.1.2018 | HN- 31AA | CPV-2c | 52.6 | 20.2 | North | 3.5 M | F | N/A | N/A |
| 20.1.2018 | HN- 32AA | CPV-2c | 52.4 | 22.8 | North | 2 M | F | N/A | N/A |
| 21.1.2018 | HN-37AA | CPV-2c | 52.6 | 17.7 | North | 3M | F | N/A | N/A |
| 23.1.2018 | HN- 34AA | CPV-2c | 52.5 | 19.4 | North | 3 M | F | N/A | N/A |
| 27.1.2018 | HN- 29AA | CPV-2c | 52.7 | 18.3 | North | 5 M | M | N/A | N/A |
| 29.1.2018 | HN- 27AA | CPV-2c | 52.8 | 16.7 | North | 3 M | M | N/A | N/A |
| 31.1.2018 | HN- 21AA | CPV-2c | 52.9 | 12.1 | North | N/A | M | N/A | N/A |
| 31.1.2018 | HN- 28AA | CPV-2c | 52.6 | 15.7 | North | N/A | N/A | N/A | N/A |
| 5.2.2018 | HN- 26AA | CPV-2c | 52.7 | 17.3 | North | 10 M | N/A | N/A | N/A |
| 5.2.2018 | HN- 30AA | CPV-2c | 52.9 | 14.6 | North | 2 M | M | N/A | N/A |

^a^N/A: not available.
